# New records of *Orussus minutus* Middlekauff, 1983 (Hymenoptera: Orussidae) represent a significant western range expansion

**DOI:** 10.3897/BDJ.3.e5793

**Published:** 2015-08-31

**Authors:** Michael Joseph Skvarla, Amber Tripodi, Allen Szalanski, Ashley Dowling

**Affiliations:** ‡ University of Arkansas, Fayetteville, United States of America; § USDA ARS Pollinating Insects Research Unit, Logan, United States of America

**Keywords:** Symphyta, Range expansion, Buffalo National River, new state record

## Abstract

**Background:**

*Orussus minutus* is an uncommonly collected parasitoid sawfly known from the eastern United States.

**New information:**

We report specimens *Orussus minutus* Middlekauff, 1983, from Arkansas, Iowa, Minnesota, and Manitoba, which represent new state and province records and significantly expand the known range of the species west from previous records; provide collection information for unpublished specimens housed in the United States National Museum collection, which includes new state records for West Virginia and Michigan; and report two specimens housed in the Biological Museum at Lund University that represent new state records for Connecticut.

## Introduction

Orussidae have long interested entomologists because of their parasitoid larvae, which are unique among non-apocritan Hymenoptera, phylogenetically important position between basal Hymenoptera ("Symphyta") and Apocrita, and because they are rarely collected (Middlekauff 1983, Pesarini and Turrisi 2003, Vilhelmsen 2003).

Middlekauff (1983) provided an excellent review of the literature concerning the feeding biology and hosts of orussid larvae. Briefly summarized, a number of authors reported orussid larvae develop in wood (Harrington 1887a, Konow 1902, Gaulle 1906) and associate with beetle and sawfly larvae (Wachtl 1882, Rudow 1909). Harrington (1887b) first hypothesized that orussid larvae may be parasitoids, though he considered it more likely they fed on wood. Rohwer (1912) and Burke (1918) provided convincing evidence that orussids are parasitoids as they reported *Orussus* larvae pupating in old cerambycid larval galleries and attacking buprestid larvae. Subsequent authors investigated oviposition behavior and larval feeding; they found that adult female orussids deposit eggs into frass-filled galleries of and directly onto larvae of wood-boring Coleoptera and Hymenoptera and that larval orussids feed upon those larvae (Cooper 1953, Rawlings 1957, Powell and Turner 1975). Currently, Orussidae are known or suspected to parasitize Buprestidae, Cerambycidae, Siricidae, and Xiphydriidae (Table 1).

Ashmead (1896) published the first phylogenetic hypothesis of Hymenoptera and placed Oryssidae (=Orussidae) transitionally between sawflies and other Hymenoptera. Recent phylogenetic analyses of morphological characters (Rasnitsyn 1988, Vilhelmsen 1997, Vilhelmsen 2000, Vilhelmsen 2001, Ronquist et al. 1999, Schulmeister 2003b), large molecular datasets and combined molecular and morphological datasets (Schulmeister 2003b, Heraty et al. 2011, Sharkey et al. 2011) have corroborated the placement of Orussidae (and Paroryssidae when fossil taxa are included) as sister to Apocrita. For relationships within Orussidae, the most robust phylogenetic analysis was produced by Vilhelmsen (2003). His analysis recovered most genera as monophyletic, though Vilhelmsen abandoned the use of subfamilies and tribes, as “[e]nforcing a strictly cladistics classification at these levels would require recognition of many redundant taxa without enhancing the information content”.

Orussidae are uncommonly collected and rare in collections. For example, despite a cumulative 25,000 trapping hours (314 separate 1–2 week collection events) using Malaise traps over the last five years by the authors around Arkansas, no additional specimens beyond the three reported herein were captured with this trapping method and David Smith (USDA, SEL), who has had success collecting orussids in Malaise traps (e.g., Smith 2006, Smith 2008, Barrows and Smith 2014), has only collected 33 specimens of *O. minutus* in 35 years of collecting with an average of 15 Malaise traps set per year (David R. Smith, pers. comm. 18 August 2015). Additionally, new species continue to be described, even in heavily collected areas such as California (e.g., Vilhelmsen 2005, Blank et al. 2010, Vilhelmsen et al. 2014). Several species are known only from one or a few localities and specimens and the known ranges of many species continue to expand as new specimens are collected (Ahnlund and Ronquist 2001, Vilhelmsen and Smith 2002, Pesarini and Turrisi 2003, Pesarini and Turrisi 2006, Choi and Suh 2011).

*Orussus* is represented five species in North America north of Mexico: *O. occidentalis* (Cresson, 1879) has been reported from Southern British Columbia east to Ontario, south in the western United States to southern California, Nevada, and New Mexico; *O. thoracicus* (Ashmead, 1898) has been reported from Colorado, Washington, Oregon, and California; *O. sayii* (Westwood, 1835) has been reported from Ontario south to Louisiana, west to Indiana; *O. terminalis* (Newman, 1838) has been reported from New England and Ontario west to Iowa and Illinois, south to Maryland; and *O. minutus* (Middlekauff, 1983) has been reported from New York to Georgia west to Illinois (Middlekauff 1983, Vilhelmsen 2003, Blank et al. 2010, Vilhelmsen et al. 2013).

## Materials and methods

Two orussids (1 male, 1 female) were collected along the Buffalo National River in the lower collector of an aerial SLAM (sea-land-air-Malaise) trap (MegaView Science Co., Ltd., Taichung, Taiwan) and a black multifunnel trap (ChemTica International, S.A., Heredia, Costa Rica); a third specimen (1 female) was collected via aerial netting in the Kessler Mountain Reserve. Both localities are mixed secondary deciduous forest dominated by oak and hickory that were logged approximately 80–100 years ago. Specimens were identified to species using published keys (Middlekauff 1983, Vilhelmsen et al. 2014) and have been deposited in the University of Arkansas Arthropod Museum.

Stereomicrographs of the Arkansas specimens were taken with a Cannon EOS 40D camera (Tokyo, Japan) attached using a Diagnostic Instruments DD20NLT 2.0X camera mount (Sterling Heights, Michigan, USA) to a Nikon SMZ1500 stereomicroscope (Tokyo, Japan). The microgrpahs were processed and final plates arranged in Adobe Illustrator (San Jose, California, USA).

DNA of one Arkansas specimen (MS 13-0413-047, #138295) was sequenced for comparison with previously characterized *Orussus*. Genomic DNA was extracted from a single mid-leg using the Qiagen DNeasy Tissue kit (Qiagen, Inc., Valencia, California), following manufacturer’s instructions. PCR was conducted using the primers LR-J-13017 (5’- TTACGCTGTTATCCTAA-3’) and LR-N-13398 (5’- CACCTGTTTAACAAAAACAT-3’) (Kambhampati and Smith 1995), which amplify an approximately 415 bp portion of the 16S rRNA region of the mitochondrial genome. Reaction conditions were 94°C for 2 min, followed by 40 cycles of 94°C for 45 s, 48°C for 1 min, and 72°C for 1 min, with a final 5 min extension step at 72°C. Amplified DNA was purified, concentrated with PES 30k centrifugal filter devices (VWR, Radnor, PA) and sent for direct sequencing in both directions (Eurofins MWG Operon, Huntsville, Alabama).

David R. Smith kindly provided label information for specimens housed in the United States National Museum; previously unpublished specimens are reported herein. Additional unpublished specimens were found by searching the databased collection of Lund University Biological Museum (Lund University 2015), BugGuide (Hatfield 2008, Alexander 2011, Liberta 2014, Zhang 2014), and Flickr (King 2014).

**Figure 3.**
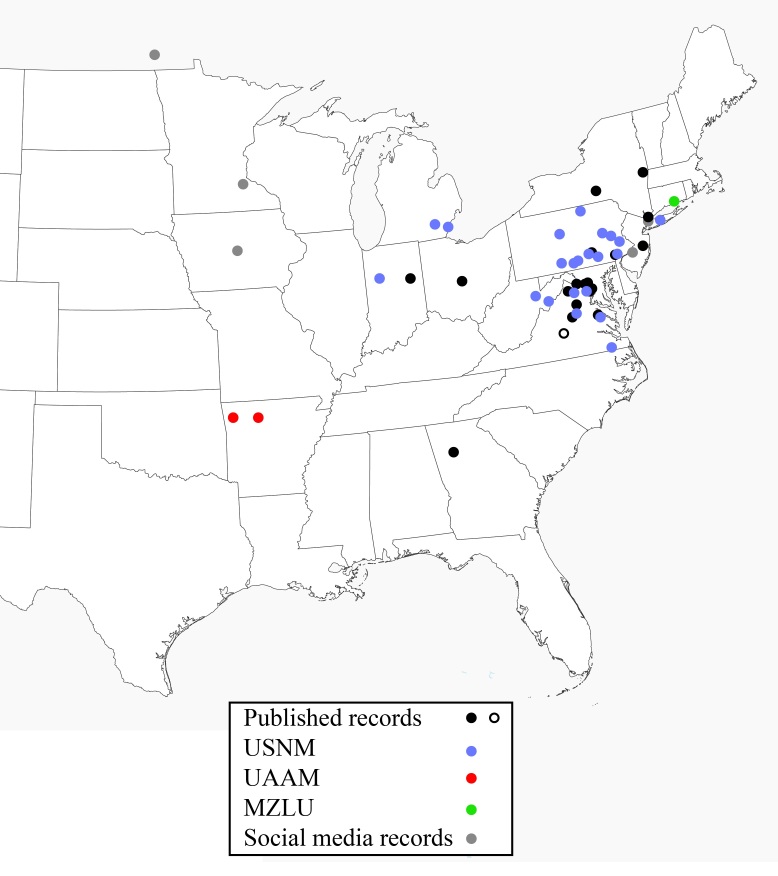
Known range of *Orussus minutus*. Solid circles represent collection localities, open circles represent state records lacking additional locality data.

Published locality data for Figure 3 was compiled from Cooper (1953), Middlekauff (1983), Smith (2006), Barrows and Smith (2014).

Institution abbreviations follow Evenhuis (2015) and are as follows: United States National Museum (USNM), University of Arkansas Arthropod Museum (UAAM), Lund University, Sweden (MZLU).

## Taxon treatments

### Ourssus minutus

Middlekauff, 1983

#### Materials

- **Type status:** Other material. **Occurrence:** catalogNumber: 138295; recordedBy: Michael J Skvarla; individualCount: 1; lifeStage: adult; **Taxon:** scientificName: *Orussus minutus* Middlekauff, 1983; kingdom: Animalia; phylum: Arthropoda; class: Insecta; order: Hymenoptera; family: Orussidae; genus: Orussus; specificEpithet: minutus; scientificNameAuthorship: Middlekauff, 1983; **Location:** country: United States; countryCode: US; stateProvince: Arkansas; county: Newton; locality: Buffalo National River, Steel Creek; locationRemarks: 80-100 year old mature second-growth Eastern mixed deciduous forest dominated by oak (Quercus) and hickory (Carya); verbatimCoordinates: 36°02.218' N, 93°20.439 W; decimalLatitude: 36.036967; decimalLongitude: -93.34065; georeferenceProtocol: GPS; **Identification:** identifiedBy: Michael J. Skvarla; dateIdentified: 2014; **Event:** samplingProtocol: black Lindgren multifunnel trap; eventDate: 201313-4-13; **Record Level:** language: en; collectionID: MS 13-0413-047; institutionCode: UAAM; basisOfRecord: PreservedSpecimen
- **Type status:** Other material. **Occurrence:** catalogNumber: 138296; recordedBy: Michael J Skvarla; individualCount: 1; lifeStage: adult; **Taxon:** scientificName: *Orussus minutus* Middlekauff, 1983; kingdom: Animalia; phylum: Arthropoda; class: Insecta; order: Hymenoptera; family: Orussidae; genus: Orussus; specificEpithet: minutus; scientificNameAuthorship: Middlekauff, 1983; **Location:** country: United States; countryCode: US; stateProvince: Arkansas; county: Newton; locality: Buffalo National River, Steel Creek; locationRemarks: 80-100 year old mature second-growth Eastern mixed deciduous forest dominated by oak (Quercus) and hickory (Carya); verbatimCoordinates: 36°02.314' N, 93°20.425 W; decimalLatitude: 36.038567; decimalLongitude: -93.34041; georeferenceProtocol: GPS; **Identification:** identifiedBy: Michael J. Skvarla; dateIdentified: 2014; **Event:** samplingProtocol: SLAM canopy trap, lower collector; eventDate: 201313-4-13; **Record Level:** language: en; collectionID: MS 13-0413-060; institutionCode: UAAM; basisOfRecord: PreservedSpecimen
- **Type status:** Other material. **Occurrence:** recordedBy: Amber Tripodi; individualCount: 1; lifeStage: adult; **Taxon:** scientificName: *Orussus minutus* Middlekauff, 1983; kingdom: Animalia; phylum: Arthropoda; class: Insecta; order: Hymenoptera; family: Orussidae; genus: Orussus; specificEpithet: minutus; scientificNameAuthorship: Middlekauff, 1983; **Location:** country: United States; countryCode: US; stateProvince: Arkansas; county: Washington; locality: Fayetteville, Kessler Mountain Reserve, Wino Trail; locationRemarks: 80-100 year old mature second-growth Eastern mixed deciduous forest dominated by oak (Quercus) and hickory (Carya); verbatimCoordinates: 36°02'19.45" N, 94°13'01.98" W; decimalLatitude: 36.038611; decimalLongitude: -94.216944; georeferenceProtocol: GoogleEarth; **Identification:** identifiedBy: Michael J. Skvarla; dateIdentified: 2014; **Event:** samplingProtocol: hand collected with net; eventDate: 41755.00; **Record Level:** language: en; institutionCode: UAAM; basisOfRecord: PreservedSpecimen
- **Type status:** Other material. **Occurrence:** recordedBy: T. P. Nuhn; individualCount: 1; lifeStage: adult; **Taxon:** scientificName: *Orussus minutus* Middlekauff, 1983; kingdom: Animalia; phylum: Arthropoda; class: Insecta; order: Hymenoptera; family: Orussidae; genus: Orussus; specificEpithet: minutus; scientificNameAuthorship: Middlekauff, 1983; **Location:** country: United States; countryCode: US; stateProvince: Virginia; county: Warren; locality: Skyland Estates; locationRemarks: 4 km NNW of Linden; georeferenceProtocol: label; **Identification:** identifiedBy: David R. Smith; **Event:** samplingProtocol: Malaise trap; eventDate: 1985-4-20/1985-4-27; **Record Level:** language: en; institutionCode: USNM; basisOfRecord: PreservedSpecimen
- **Type status:** Other material. **Occurrence:** recordedBy: T. P. Nuhn; individualCount: 1; lifeStage: adult; **Taxon:** scientificName: *Orussus minutus* Middlekauff, 1983; kingdom: Animalia; phylum: Arthropoda; class: Insecta; order: Hymenoptera; family: Orussidae; genus: Orussus; specificEpithet: minutus; scientificNameAuthorship: Middlekauff, 1983; **Location:** country: United States; countryCode: US; stateProvince: Virginia; county: Warren; locality: Skyland Estates; locationRemarks: 4 km NNW of Linden; georeferenceProtocol: label; **Identification:** identifiedBy: David R. Smith; **Event:** samplingProtocol: Malaise trap; eventDate: 1996-4-27/1996-5-12; **Record Level:** language: en; institutionCode: USNM; basisOfRecord: PreservedSpecimen
- **Type status:** Other material. **Occurrence:** recordedBy: P. J. Spangler; individualCount: 1; lifeStage: adult; **Taxon:** scientificName: *Orussus minutus* Middlekauff, 1983; kingdom: Animalia; phylum: Arthropoda; class: Insecta; order: Hymenoptera; family: Orussidae; genus: Orussus; specificEpithet: minutus; scientificNameAuthorship: Middlekauff, 1983; **Location:** country: United States; countryCode: US; stateProvince: Virginia; locality: Great Dismal Swamp National Wildlife Refuge; georeferenceProtocol: label; **Identification:** identifiedBy: David R. Smith; **Event:** samplingProtocol: Malaise trap; eventDate: 1965-4-16/1965-4-17; **Record Level:** language: en; institutionCode: USNM; basisOfRecord: PreservedSpecimen
- **Type status:** Other material. **Occurrence:** recordedBy: J. Kloke & D. R. Smith; individualCount: 1; lifeStage: adult; **Taxon:** scientificName: *Orussus minutus* Middlekauff, 1983; kingdom: Animalia; phylum: Arthropoda; class: Insecta; order: Hymenoptera; family: Orussidae; genus: Orussus; specificEpithet: minutus; scientificNameAuthorship: Middlekauff, 1983; **Location:** country: United States; countryCode: US; stateProvince: Virginia; county: Louisa; locationRemarks: 4 mi south of Cuckoo; georeferenceProtocol: label; **Identification:** identifiedBy: David R. Smith; **Event:** samplingProtocol: Malaise trap; eventDate: 1989-4-26/1989-5-12; **Record Level:** language: en; institutionCode: USNM; basisOfRecord: PreservedSpecimen
- **Type status:** Other material. **Occurrence:** recordedBy: J. Kloke & D. R. Smith; individualCount: 1; lifeStage: adult; **Taxon:** scientificName: *Orussus minutus* Middlekauff, 1983; kingdom: Animalia; phylum: Arthropoda; class: Insecta; order: Hymenoptera; family: Orussidae; genus: Orussus; specificEpithet: minutus; scientificNameAuthorship: Middlekauff, 1983; **Location:** country: United States; countryCode: US; stateProvince: Virginia; county: Louisa; locationRemarks: 4 mi south of Cuckoo; georeferenceProtocol: label; **Identification:** identifiedBy: David R. Smith; **Event:** samplingProtocol: Malaise trap; eventDate: 1989-5-27/1989-6-7; **Record Level:** language: en; institutionCode: USNM; basisOfRecord: PreservedSpecimen
- **Type status:** Other material. **Occurrence:** recordedBy: J. Kloke & D. R. Smith; individualCount: 1; lifeStage: adult; **Taxon:** scientificName: *Orussus minutus* Middlekauff, 1983; kingdom: Animalia; phylum: Arthropoda; class: Insecta; order: Hymenoptera; family: Orussidae; genus: Orussus; specificEpithet: minutus; scientificNameAuthorship: Middlekauff, 1983; **Location:** country: United States; countryCode: US; stateProvince: Virginia; county: Louisa; locationRemarks: 4 mi south of Cuckoo; georeferenceProtocol: label; **Identification:** identifiedBy: David R. Smith; **Event:** samplingProtocol: Malaise trap; eventDate: 1988-3-19/1988-4-11; **Record Level:** language: en; institutionCode: USNM; basisOfRecord: PreservedSpecimen
- **Type status:** Other material. **Occurrence:** recordedBy: D. R. Smith; individualCount: 1; lifeStage: adult; **Taxon:** scientificName: *Orussus minutus* Middlekauff, 1983; kingdom: Animalia; phylum: Arthropoda; class: Insecta; order: Hymenoptera; family: Orussidae; genus: Orussus; specificEpithet: minutus; scientificNameAuthorship: Middlekauff, 1983; **Location:** country: United States; countryCode: US; stateProvince: Virginia; county: Fairfax; locality: Holmes Run; locationRemarks: ~1/4 mi NW jct. Gallows Rd & I-495; verbatimCoordinates: 38°50’N, 77°12’W; georeferenceProtocol: label; **Identification:** identifiedBy: David R. Smith; **Event:** samplingProtocol: Malaise trap; eventDate: 1990-4-22/1990-4-28; **Record Level:** language: en; institutionCode: USNM; basisOfRecord: PreservedSpecimen
- **Type status:** Other material. **Occurrence:** recordedBy: D. R. Smith; individualCount: 1; lifeStage: adult; **Taxon:** scientificName: *Orussus minutus* Middlekauff, 1983; kingdom: Animalia; phylum: Arthropoda; class: Insecta; order: Hymenoptera; family: Orussidae; genus: Orussus; specificEpithet: minutus; scientificNameAuthorship: Middlekauff, 1983; **Location:** country: United States; countryCode: US; stateProvince: Virginia; county: Fairfax; locality: Holmes Run; locationRemarks: ~1/4 mi NW jct. Gallows Rd & I-496; verbatimCoordinates: 38°50’N, 77°12’W; georeferenceProtocol: label; **Identification:** identifiedBy: David R. Smith; **Event:** samplingProtocol: Malaise trap; eventDate: 1990-3-11/1990-30-17; **Record Level:** language: en; institutionCode: USNM; basisOfRecord: PreservedSpecimen
- **Type status:** Other material. **Occurrence:** recordedBy: D. R. Smith; individualCount: 1; lifeStage: adult; **Taxon:** scientificName: *Orussus minutus* Middlekauff, 1983; kingdom: Animalia; phylum: Arthropoda; class: Insecta; order: Hymenoptera; family: Orussidae; genus: Orussus; specificEpithet: minutus; scientificNameAuthorship: Middlekauff, 1983; **Location:** country: United States; countryCode: US; stateProvince: Virginia; county: Fairfax; locality: Holmes Run; locationRemarks: ~1/4 mi NW jct. Gallows Rd & I-497; verbatimCoordinates: 38°50’N, 77°12’W; georeferenceProtocol: label; **Identification:** identifiedBy: David R. Smith; **Event:** samplingProtocol: Malaise trap; eventDate: 2008-4-13/2008-4-19; **Record Level:** language: en; institutionCode: USNM; basisOfRecord: PreservedSpecimen
- **Type status:** Other material. **Occurrence:** recordedBy: D. R. Smith; individualCount: 1; lifeStage: adult; **Taxon:** scientificName: *Orussus minutus* Middlekauff, 1983; kingdom: Animalia; phylum: Arthropoda; class: Insecta; order: Hymenoptera; family: Orussidae; genus: Orussus; specificEpithet: minutus; scientificNameAuthorship: Middlekauff, 1983; **Location:** country: United States; countryCode: US; stateProvince: Virginia; county: Clarke; locality: University of Virginia Blandy Experiment Farm; locationRemarks: 2 mi south of Boyce; verbatimCoordinates: 39°05’N, 78°10’W; georeferenceProtocol: label; **Identification:** identifiedBy: David R. Smith; **Event:** samplingProtocol: Malaise trap; eventDate: 1992-5-2/1992-5-16; **Record Level:** language: en; institutionCode: USNM; basisOfRecord: PreservedSpecimen
- **Type status:** Other material. **Occurrence:** recordedBy: D. R. Smith; individualCount: 2; lifeStage: adult; **Taxon:** scientificName: *Orussus minutus* Middlekauff, 1983; kingdom: Animalia; phylum: Arthropoda; class: Insecta; order: Hymenoptera; family: Orussidae; genus: Orussus; specificEpithet: minutus; scientificNameAuthorship: Middlekauff, 1983; **Location:** country: United States; countryCode: US; stateProvince: Virginia; county: Clarke; locality: University of Virginia Blandy Experiment Farm; locationRemarks: 2 mi south of Boyce; verbatimCoordinates: 39°05’N, 78°10’W; georeferenceProtocol: label; **Identification:** identifiedBy: David R. Smith; **Event:** samplingProtocol: Malaise trap; eventDate: 1994-4-16/1994-4-28; **Record Level:** language: en; institutionCode: USNM; basisOfRecord: PreservedSpecimen
- **Type status:** Other material. **Occurrence:** recordedBy: D. R. Smith; individualCount: 1; lifeStage: adult; **Taxon:** scientificName: *Orussus minutus* Middlekauff, 1983; kingdom: Animalia; phylum: Arthropoda; class: Insecta; order: Hymenoptera; family: Orussidae; genus: Orussus; specificEpithet: minutus; scientificNameAuthorship: Middlekauff, 1983; **Location:** country: United States; countryCode: US; stateProvince: Virginia; county: Clarke; locality: University of Virginia Blandy Experiment Farm; locationRemarks: 2 mi south of Boyce; verbatimCoordinates: 39°05’N, 78°10’W; georeferenceProtocol: label; **Identification:** identifiedBy: David R. Smith; **Event:** samplingProtocol: Malaise trap; eventDate: 1994-4-16/1994-4-28; **Record Level:** language: en; institutionCode: USNM; basisOfRecord: PreservedSpecimen
- **Type status:** Other material. **Occurrence:** recordedBy: D. R. Smith; individualCount: 1; lifeStage: adult; **Taxon:** scientificName: *Orussus minutus* Middlekauff, 1983; kingdom: Animalia; phylum: Arthropoda; class: Insecta; order: Hymenoptera; family: Orussidae; genus: Orussus; specificEpithet: minutus; scientificNameAuthorship: Middlekauff, 1983; **Location:** country: United States; countryCode: US; stateProvince: Virginia; county: Essex; locationRemarks: 1 mi southeast of Dunnsville; verbatimCoordinates: 37°52’N, 76°48’W; georeferenceProtocol: label; **Identification:** identifiedBy: David R. Smith; **Event:** samplingProtocol: Malaise trap; eventDate: 1992-4-1/1992-4-16; **Record Level:** language: en; institutionCode: USNM; basisOfRecord: PreservedSpecimen
- **Type status:** Other material. **Occurrence:** recordedBy: D. R. Smith; individualCount: 1; lifeStage: adult; **Taxon:** scientificName: *Orussus minutus* Middlekauff, 1983; kingdom: Animalia; phylum: Arthropoda; class: Insecta; order: Hymenoptera; family: Orussidae; genus: Orussus; specificEpithet: minutus; scientificNameAuthorship: Middlekauff, 1983; **Location:** country: United States; countryCode: US; stateProvince: Virginia; county: Essex; locationRemarks: 1 mi southeast of Dunnsville; verbatimCoordinates: 37°52’N, 76°48’W; georeferenceProtocol: label; **Identification:** identifiedBy: David R. Smith; **Event:** samplingProtocol: Malaise trap; eventDate: 1993-5-15/1993-5-28; **Record Level:** language: en; institutionCode: USNM; basisOfRecord: PreservedSpecimen
- **Type status:** Other material. **Occurrence:** recordedBy: D. R. Smith; individualCount: 2; lifeStage: adult; **Taxon:** scientificName: *Orussus minutus* Middlekauff, 1983; kingdom: Animalia; phylum: Arthropoda; class: Insecta; order: Hymenoptera; family: Orussidae; genus: Orussus; specificEpithet: minutus; scientificNameAuthorship: Middlekauff, 1983; **Location:** country: United States; countryCode: US; stateProvince: Virginia; county: Essex; locationRemarks: 1 mi southeast of Dunnsville; verbatimCoordinates: 37°52’N, 76°48’W; georeferenceProtocol: label; **Identification:** identifiedBy: David R. Smith; **Event:** samplingProtocol: Malaise trap; eventDate: 1994-4-22/1994-5-3; **Record Level:** language: en; institutionCode: USNM; basisOfRecord: PreservedSpecimen
- **Type status:** Other material. **Occurrence:** recordedBy: D. R. Smith; individualCount: 1; lifeStage: adult; **Taxon:** scientificName: *Orussus minutus* Middlekauff, 1983; kingdom: Animalia; phylum: Arthropoda; class: Insecta; order: Hymenoptera; family: Orussidae; genus: Orussus; specificEpithet: minutus; scientificNameAuthorship: Middlekauff, 1983; **Location:** country: United States; countryCode: US; stateProvince: Virginia; county: Essex; locationRemarks: 1 mi southeast of Dunnsville; verbatimCoordinates: 37°52’N, 76°48’W; georeferenceProtocol: label; **Identification:** identifiedBy: David R. Smith; **Event:** samplingProtocol: Malaise trap; eventDate: 1994-4-22/1994-5-3; **Record Level:** language: en; institutionCode: USNM; basisOfRecord: PreservedSpecimen
- **Type status:** Other material. **Occurrence:** recordedBy: D. R. Smith; individualCount: 1; lifeStage: adult; **Taxon:** scientificName: *Orussus minutus* Middlekauff, 1983; kingdom: Animalia; phylum: Arthropoda; class: Insecta; order: Hymenoptera; family: Orussidae; genus: Orussus; specificEpithet: minutus; scientificNameAuthorship: Middlekauff, 1983; **Location:** country: United States; countryCode: US; stateProvince: Virginia; county: Essex; locationRemarks: 1 mi southeast of Dunnsville; verbatimCoordinates: 37°52’N, 76°48’W; georeferenceProtocol: label; **Identification:** identifiedBy: David R. Smith; **Event:** samplingProtocol: Malaise trap; eventDate: 1995-3-23/1995-4-11; **Record Level:** language: en; institutionCode: USNM; basisOfRecord: PreservedSpecimen
- **Type status:** Other material. **Occurrence:** recordedBy: D. R. Smith; individualCount: 1; lifeStage: adult; **Taxon:** scientificName: *Orussus minutus* Middlekauff, 1983; kingdom: Animalia; phylum: Arthropoda; class: Insecta; order: Hymenoptera; family: Orussidae; genus: Orussus; specificEpithet: minutus; scientificNameAuthorship: Middlekauff, 1983; **Location:** country: United States; countryCode: US; stateProvince: Virginia; county: Essex; locationRemarks: 1 mi southeast of Dunnsville; verbatimCoordinates: 37°52’N, 76°48’W; georeferenceProtocol: label; **Identification:** identifiedBy: David R. Smith; **Event:** samplingProtocol: Malaise trap; eventDate: 1996-4-12/1996-5-6; **Record Level:** language: en; institutionCode: USNM; basisOfRecord: PreservedSpecimen
- **Type status:** Other material. **Occurrence:** recordedBy: D. R. Smith; individualCount: 1; lifeStage: adult; **Taxon:** scientificName: *Orussus minutus* Middlekauff, 1983; kingdom: Animalia; phylum: Arthropoda; class: Insecta; order: Hymenoptera; family: Orussidae; genus: Orussus; specificEpithet: minutus; scientificNameAuthorship: Middlekauff, 1983; **Location:** country: United States; countryCode: US; stateProvince: Virginia; county: Essex; locationRemarks: 1 mi southeast of Dunnsville; verbatimCoordinates: 37°52’N, 76°48’W; georeferenceProtocol: label; **Identification:** identifiedBy: David R. Smith; **Event:** samplingProtocol: Malaise trap; eventDate: 1996-5-7/1996-5-17; **Record Level:** language: en; institutionCode: USNM; basisOfRecord: PreservedSpecimen
- **Type status:** Other material. **Occurrence:** recordedBy: D. R. Smith; individualCount: 1; lifeStage: adult; **Taxon:** scientificName: *Orussus minutus* Middlekauff, 1983; kingdom: Animalia; phylum: Arthropoda; class: Insecta; order: Hymenoptera; family: Orussidae; genus: Orussus; specificEpithet: minutus; scientificNameAuthorship: Middlekauff, 1983; **Location:** country: United States; countryCode: US; stateProvince: Virginia; county: Essex; locationRemarks: 1 mi southeast of Dunnsville; verbatimCoordinates: 37°52’N, 76°48’W; georeferenceProtocol: label; **Identification:** identifiedBy: David R. Smith; **Event:** samplingProtocol: Malaise trap; eventDate: 1999-3-6/1999-3-20; **Record Level:** language: en; institutionCode: USNM; basisOfRecord: PreservedSpecimen
- **Type status:** Other material. **Occurrence:** recordedBy: D. R. Smith; individualCount: 1; lifeStage: adult; **Taxon:** scientificName: *Orussus minutus* Middlekauff, 1983; kingdom: Animalia; phylum: Arthropoda; class: Insecta; order: Hymenoptera; family: Orussidae; genus: Orussus; specificEpithet: minutus; scientificNameAuthorship: Middlekauff, 1983; **Location:** country: United States; countryCode: US; stateProvince: Virginia; county: Essex; locationRemarks: 1 mi southeast of Dunnsville; verbatimCoordinates: 37°52’N, 76°48’W; georeferenceProtocol: label; **Identification:** identifiedBy: David R. Smith; **Event:** samplingProtocol: Malaise trap; eventDate: 1999-4-3/1999-4-19; **Record Level:** language: en; institutionCode: USNM; basisOfRecord: PreservedSpecimen
- **Type status:** Other material. **Occurrence:** recordedBy: D. R. Smith; individualCount: 1; lifeStage: adult; **Taxon:** scientificName: *Orussus minutus* Middlekauff, 1983; kingdom: Animalia; phylum: Arthropoda; class: Insecta; order: Hymenoptera; family: Orussidae; genus: Orussus; specificEpithet: minutus; scientificNameAuthorship: Middlekauff, 1983; **Location:** country: United States; countryCode: US; stateProvince: Virginia; county: Essex; locationRemarks: 1 mi southeast of Dunnsville; verbatimCoordinates: 37°52’N, 76°48’W; georeferenceProtocol: label; **Identification:** identifiedBy: David R. Smith; **Event:** samplingProtocol: Malaise trap; eventDate: 1999-4-3/1999-4-19; **Record Level:** language: en; institutionCode: USNM; basisOfRecord: PreservedSpecimen
- **Type status:** Other material. **Occurrence:** recordedBy: D. R. Smith; individualCount: 1; lifeStage: adult; **Taxon:** scientificName: *Orussus minutus* Middlekauff, 1983; kingdom: Animalia; phylum: Arthropoda; class: Insecta; order: Hymenoptera; family: Orussidae; genus: Orussus; specificEpithet: minutus; scientificNameAuthorship: Middlekauff, 1983; **Location:** country: United States; countryCode: US; stateProvince: Virginia; county: Essex; locationRemarks: 1 mi southeast of Dunnsville; verbatimCoordinates: 37°52’N, 76°48’W; georeferenceProtocol: label; **Identification:** identifiedBy: David R. Smith; **Event:** samplingProtocol: Malaise trap; eventDate: 1999-5-6/1999-5-20; **Record Level:** language: en; institutionCode: USNM; basisOfRecord: PreservedSpecimen
- **Type status:** Other material. **Occurrence:** recordedBy: D. R. Smith; individualCount: 1; lifeStage: adult; **Taxon:** scientificName: *Orussus minutus* Middlekauff, 1983; kingdom: Animalia; phylum: Arthropoda; class: Insecta; order: Hymenoptera; family: Orussidae; genus: Orussus; specificEpithet: minutus; scientificNameAuthorship: Middlekauff, 1983; **Location:** country: United States; countryCode: US; stateProvince: Virginia; county: Fairfax; locality: Great Falls Park; verbatimCoordinates: 38°59.4’N, 77°15.26’W; georeferenceProtocol: label; **Identification:** identifiedBy: David R. Smith; **Event:** samplingProtocol: Malaise trap; eventDate: 2007-4-19/2007-5-2; **Record Level:** language: en; institutionCode: USNM; basisOfRecord: PreservedSpecimen
- **Type status:** Other material. **Occurrence:** recordedBy: D. R. Smith; individualCount: 1; lifeStage: adult; **Taxon:** scientificName: *Orussus minutus* Middlekauff, 1983; kingdom: Animalia; phylum: Arthropoda; class: Insecta; order: Hymenoptera; family: Orussidae; genus: Orussus; specificEpithet: minutus; scientificNameAuthorship: Middlekauff, 1983; **Location:** country: United States; countryCode: US; stateProvince: West Virginia; county: Hardy; locationRemarks: 3 mi northeast of Mathias; verbatimCoordinates: 38°55’N, 78°49’W; georeferenceProtocol: label; **Identification:** identifiedBy: David R. Smith; **Event:** samplingProtocol: Malaise trap; eventDate: 2000-5-1/2000-5-15; **Record Level:** language: en; institutionCode: USNM; basisOfRecord: PreservedSpecimen
- **Type status:** Other material. **Occurrence:** recordedBy: D. R. Smith; individualCount: 1; lifeStage: adult; **Taxon:** scientificName: *Orussus minutus* Middlekauff, 1983; kingdom: Animalia; phylum: Arthropoda; class: Insecta; order: Hymenoptera; family: Orussidae; genus: Orussus; specificEpithet: minutus; scientificNameAuthorship: Middlekauff, 1983; **Location:** country: United States; countryCode: US; stateProvince: West Virginia; county: Hardy; locationRemarks: 3 mi northeast of Mathias; verbatimCoordinates: 38°55’N, 78°49’W; georeferenceProtocol: label; **Identification:** identifiedBy: David R. Smith; **Event:** samplingProtocol: Malaise trap; eventDate: 2001-4-1/2001-5-14; **Record Level:** language: en; institutionCode: USNM; basisOfRecord: PreservedSpecimen
- **Type status:** Other material. **Occurrence:** recordedBy: D. R. Smith; individualCount: 1; lifeStage: adult; **Taxon:** scientificName: *Orussus minutus* Middlekauff, 1983; kingdom: Animalia; phylum: Arthropoda; class: Insecta; order: Hymenoptera; family: Orussidae; genus: Orussus; specificEpithet: minutus; scientificNameAuthorship: Middlekauff, 1983; **Location:** country: United States; countryCode: US; stateProvince: West Virginia; county: Hardy; locationRemarks: 3 mi northeast of Mathias; verbatimCoordinates: 38°55’N, 78°49’W; georeferenceProtocol: label; **Identification:** identifiedBy: David R. Smith; **Event:** samplingProtocol: Malaise trap; eventDate: 2007-5-4/2007-5-21; **Record Level:** language: en; institutionCode: USNM; basisOfRecord: PreservedSpecimen
- **Type status:** Other material. **Occurrence:** recordedBy: D. R. Smith; individualCount: 1; lifeStage: adult; **Taxon:** scientificName: *Orussus minutus* Middlekauff, 1983; kingdom: Animalia; phylum: Arthropoda; class: Insecta; order: Hymenoptera; family: Orussidae; genus: Orussus; specificEpithet: minutus; scientificNameAuthorship: Middlekauff, 1983; **Location:** country: United States; countryCode: US; stateProvince: West Virginia; county: Hardy; locationRemarks: 3 mi northeast of Mathias; verbatimCoordinates: 38°55’N, 78°49’W; georeferenceProtocol: label; **Identification:** identifiedBy: David R. Smith; **Event:** samplingProtocol: Malaise trap; eventDate: 2007-5-22/2007-6-7; **Record Level:** language: en; institutionCode: USNM; basisOfRecord: PreservedSpecimen
- **Type status:** Other material. **Occurrence:** recordedBy: D. R. Smith; individualCount: 1; lifeStage: adult; **Taxon:** scientificName: *Orussus minutus* Middlekauff, 1983; kingdom: Animalia; phylum: Arthropoda; class: Insecta; order: Hymenoptera; family: Orussidae; genus: Orussus; specificEpithet: minutus; scientificNameAuthorship: Middlekauff, 1983; **Location:** country: United States; countryCode: US; stateProvince: West Virginia; county: Hardy; locationRemarks: 3 mi northeast of Mathias; verbatimCoordinates: 38°55’N, 78°49’W; georeferenceProtocol: label; **Identification:** identifiedBy: David R. Smith; **Event:** samplingProtocol: Malaise trap; eventDate: 2008-5-30/2008-6-17; **Record Level:** language: en; institutionCode: USNM; basisOfRecord: PreservedSpecimen
- **Type status:** Other material. **Occurrence:** recordedBy: E. M. Barrows; individualCount: 1; lifeStage: adult; **Taxon:** scientificName: *Orussus minutus* Middlekauff, 1983; kingdom: Animalia; phylum: Arthropoda; class: Insecta; order: Hymenoptera; family: Orussidae; genus: Orussus; specificEpithet: minutus; scientificNameAuthorship: Middlekauff, 1983; **Location:** country: United States; countryCode: US; stateProvince: West Virginia; county: Tucker; locality: Fernow Experimental Forest; verbatimCoordinates: 39°03’N, 79°40’W; georeferenceProtocol: label; **Event:** samplingProtocol: Malaise trap; eventDate: 1993-4-30/1993-5-10; **Record Level:** language: en; institutionCode: USNM; basisOfRecord: PreservedSpecimen
- **Type status:** Other material. **Occurrence:** recordedBy: K. V. Krombein; individualCount: 5; lifeStage: adult; behavior: specimens taken on trunk of dead, standing, barked samplings, trunk diam. 2"; associatedReferences: Smith, D.R. 2008. Hymenoptera (Insecta) of Plummers Island, Maryland: Symphyta and selected families of Apocrita. Bulletin of the Biological Society of Washington, 15(1): 160–167; **Taxon:** scientificName: *Orussus minutus* Middlekauff, 1983; kingdom: Animalia; phylum: Arthropoda; class: Insecta; order: Hymenoptera; family: Orussidae; genus: Orussus; specificEpithet: minutus; scientificNameAuthorship: Middlekauff, 1983; **Location:** country: United States; countryCode: US; stateProvince: Maryland; county: Montgomery; locality: Plummers Island; georeferenceProtocol: label; **Identification:** identifiedBy: David R. Smith; **Event:** samplingProtocol: hand collected with net; eventDate: 1971-4-11; **Record Level:** language: en; institutionCode: USNM; basisOfRecord: PreservedSpecimen
- **Type status:** Other material. **Occurrence:** recordedBy: Geo. Steyskal; individualCount: 1; lifeStage: adult; **Taxon:** scientificName: *Orussus minutus* Middlekauff, 1983; kingdom: Animalia; phylum: Arthropoda; class: Insecta; order: Hymenoptera; family: Orussidae; genus: Orussus; specificEpithet: minutus; scientificNameAuthorship: Middlekauff, 1983; **Location:** country: United States; countryCode: US; stateProvince: Michigan; county: Wayne; locality: Grosse Ile; georeferenceProtocol: label; **Event:** eventDate: 1957-5-25; **Record Level:** language: en; institutionCode: USNM; basisOfRecord: PreservedSpecimen
- **Type status:** Other material. **Occurrence:** recordedBy: R. W. Carlson; individualCount: 1; lifeStage: adult; **Taxon:** scientificName: *Orussus minutus* Middlekauff, 1983; kingdom: Animalia; phylum: Arthropoda; class: Insecta; order: Hymenoptera; family: Orussidae; genus: Orussus; specificEpithet: minutus; scientificNameAuthorship: Middlekauff, 1983; **Location:** country: United States; countryCode: US; stateProvince: Michigan; county: Washtenaw; georeferenceProtocol: label; **Event:** eventDate: 1967-6-10; **Record Level:** language: en; institutionCode: USNM; basisOfRecord: PreservedSpecimen
- **Type status:** Other material. **Occurrence:** recordedBy: M. & N. Deyrup; individualCount: 1; lifeStage: adult; behavior: collected in flight; **Taxon:** scientificName: *Orussus minutus* Middlekauff, 1983; kingdom: Animalia; phylum: Arthropoda; class: Insecta; order: Hymenoptera; family: Orussidae; genus: Orussus; specificEpithet: minutus; scientificNameAuthorship: Middlekauff, 1983; **Location:** country: United States; countryCode: US; stateProvince: Indiana; county: Tippecanoe; locality: West Lafayette; georeferenceProtocol: label; **Identification:** identifiedBy: David R. Smith; **Event:** samplingProtocol: hand collected; eventDate: 1970-5-5; **Record Level:** language: en; institutionCode: USNM; basisOfRecord: PreservedSpecimen
- **Type status:** Other material. **Occurrence:** recordedBy: M. & N. Deyrup; individualCount: 1; lifeStage: adult; behavior: collected from branches of Acer saccharum; associatedReferences: Deyrup, M.A. 1984. A maple wood wasp, Xiphydria maculate, and its insect enemies (Hymenoptera: Xiphydriidae). Great Lakes Entomologist, 17: 17–28. [referred to as "Orussus sp."]; **Taxon:** scientificName: *Orussus minutus* Middlekauff, 1983; kingdom: Animalia; phylum: Arthropoda; class: Insecta; order: Hymenoptera; family: Orussidae; genus: Orussus; specificEpithet: minutus; scientificNameAuthorship: Middlekauff, 1983; **Location:** country: United States; countryCode: US; stateProvince: Indiana; county: Tippecanoe; locality: West Lafayette; georeferenceProtocol: label; **Identification:** identifiedBy: David R. Smith; **Event:** eventDate: 1981-4-16; **Record Level:** language: en; institutionCode: USNM; basisOfRecord: PreservedSpecimen
- **Type status:** Other material. **Occurrence:** recordedBy: M. & N. Deyrup; individualCount: 1; lifeStage: adult; behavior: collected from branches of Acer saccharum; associatedReferences: Deyrup, M.A. 1984. A maple wood wasp, Xiphydria maculate, and its insect enemies (Hymenoptera: Xiphydriidae). Great Lakes Entomologist, 17: 17–28. [referred to as "Orussus sp."]; **Taxon:** scientificName: *Orussus minutus* Middlekauff, 1983; kingdom: Animalia; phylum: Arthropoda; class: Insecta; order: Hymenoptera; family: Orussidae; genus: Orussus; specificEpithet: minutus; scientificNameAuthorship: Middlekauff, 1983; **Location:** country: United States; countryCode: US; stateProvince: Indiana; county: Tippecanoe; locality: West Lafayette; georeferenceProtocol: label; **Identification:** identifiedBy: David R. Smith; **Event:** eventDate: 1981-4-26; **Record Level:** language: en; institutionCode: USNM; basisOfRecord: PreservedSpecimen
- **Type status:** Other material. **Occurrence:** recordedBy: Shu Ambree; individualCount: 2; lifeStage: adult; **Taxon:** scientificName: *Orussus minutus* Middlekauff, 1983; kingdom: Animalia; phylum: Arthropoda; class: Insecta; order: Hymenoptera; family: Orussidae; genus: Orussus; specificEpithet: minutus; scientificNameAuthorship: Middlekauff, 1983; **Location:** country: United States; countryCode: US; stateProvince: Pennsylvania; county: Cumberland; verbatimCoordinates: 40.22479, -76.96278; decimalLatitude: 40.22479; decimalLongitude: -76.96278; georeferenceProtocol: label; **Event:** samplingProtocol: Lindgren multifunnel trap; eventDate: 2011-5-4; **Record Level:** language: en; institutionCode: USNM; basisOfRecord: PreservedSpecimen
- **Type status:** Other material. **Occurrence:** recordedBy: Shu Ambree; individualCount: 1; lifeStage: adult; **Taxon:** scientificName: *Orussus minutus* Middlekauff, 1983; kingdom: Animalia; phylum: Arthropoda; class: Insecta; order: Hymenoptera; family: Orussidae; genus: Orussus; specificEpithet: minutus; scientificNameAuthorship: Middlekauff, 1983; **Location:** country: United States; countryCode: US; stateProvince: Pennsylvania; county: Cumberland; verbatimCoordinates: 40.22519, -76.96252; decimalLatitude: 40.22519; decimalLongitude: -76.96252; georeferenceProtocol: label; **Event:** samplingProtocol: Lindgren multifunnel trap; eventDate: 2011-5-4; **Record Level:** language: en; institutionCode: USNM; basisOfRecord: PreservedSpecimen
- **Type status:** Other material. **Occurrence:** recordedBy: Jay Bagley; individualCount: 1; lifeStage: adult; **Taxon:** scientificName: *Orussus minutus* Middlekauff, 1983; kingdom: Animalia; phylum: Arthropoda; class: Insecta; order: Hymenoptera; family: Orussidae; genus: Orussus; specificEpithet: minutus; scientificNameAuthorship: Middlekauff, 1983; **Location:** country: United States; countryCode: US; stateProvince: Pennsylvania; county: Northumberland; verbatimCoordinates: 40.87671, -76.50962; decimalLatitude: 40.87671; decimalLongitude: -76.50962; georeferenceProtocol: label; **Event:** samplingProtocol: Lindgren multifunnel trap; eventDate: 2011-6-1; **Record Level:** language: en; institutionCode: USNM; basisOfRecord: PreservedSpecimen
- **Type status:** Other material. **Occurrence:** recordedBy: Sam Louenwirth; individualCount: 1; lifeStage: adult; **Taxon:** scientificName: *Orussus minutus* Middlekauff, 1983; kingdom: Animalia; phylum: Arthropoda; class: Insecta; order: Hymenoptera; family: Orussidae; genus: Orussus; specificEpithet: minutus; scientificNameAuthorship: Middlekauff, 1983; **Location:** country: United States; countryCode: US; stateProvince: Pennsylvania; county: Lehigh; verbatimCoordinates: 40.45855, -75.473198; decimalLatitude: 40.45855; decimalLongitude: -75.473198; georeferenceProtocol: label; **Event:** samplingProtocol: Lindgren multifunnel trap; eventDate: 2012-5-31; **Record Level:** language: en; institutionCode: USNM; basisOfRecord: PreservedSpecimen
- **Type status:** Other material. **Occurrence:** recordedBy: Nathan Delp; individualCount: 1; lifeStage: adult; **Taxon:** scientificName: *Orussus minutus* Middlekauff, 1983; kingdom: Animalia; phylum: Arthropoda; class: Insecta; order: Hymenoptera; family: Orussidae; genus: Orussus; specificEpithet: minutus; scientificNameAuthorship: Middlekauff, 1983; **Location:** country: United States; countryCode: US; stateProvince: Pennsylvania; county: Bedford; verbatimCoordinates: 40.04287, -78.36906; decimalLatitude: 40.04287; decimalLongitude: -78.36906; georeferenceProtocol: label; **Event:** samplingProtocol: Lindgren multifunnel trap; eventDate: 2012-5-15; **Record Level:** language: en; institutionCode: USNM; basisOfRecord: PreservedSpecimen
- **Type status:** Other material. **Occurrence:** individualCount: 2; lifeStage: adult; **Taxon:** scientificName: *Orussus minutus* Middlekauff, 1983; kingdom: Animalia; phylum: Arthropoda; class: Insecta; order: Hymenoptera; family: Orussidae; genus: Orussus; specificEpithet: minutus; scientificNameAuthorship: Middlekauff, 1983; **Location:** country: United States; countryCode: US; stateProvince: Pennsylvania; county: Fulton; verbatimCoordinates: 40.02970, -77.637133; decimalLatitude: 40.0297; decimalLongitude: -77.637133; georeferenceProtocol: label; **Event:** samplingProtocol: Lindgren multifunnel trap; eventDate: 2014-7-8; **Record Level:** language: en; institutionCode: USNM; basisOfRecord: PreservedSpecimen
- **Type status:** Other material. **Occurrence:** recordedBy: Thea Stimmler; individualCount: 2; lifeStage: adult; **Taxon:** scientificName: *Orussus minutus* Middlekauff, 1983; kingdom: Animalia; phylum: Arthropoda; class: Insecta; order: Hymenoptera; family: Orussidae; genus: Orussus; specificEpithet: minutus; scientificNameAuthorship: Middlekauff, 1983; **Location:** country: United States; countryCode: US; stateProvince: Pennsylvania; county: Chester; verbatimCoordinates: 40.6765, -75.71953; decimalLatitude: 40.6765; decimalLongitude: -75.71953; georeferenceProtocol: label; **Event:** samplingProtocol: Lindgren multifunnel trap; eventDate: 2012-5-15; **Record Level:** language: en; institutionCode: USNM; basisOfRecord: PreservedSpecimen
- **Type status:** Other material. **Occurrence:** recordedBy: Ryan Weston; individualCount: 1; lifeStage: adult; **Taxon:** scientificName: *Orussus minutus* Middlekauff, 1983; kingdom: Animalia; phylum: Arthropoda; class: Insecta; order: Hymenoptera; family: Orussidae; genus: Orussus; specificEpithet: minutus; scientificNameAuthorship: Middlekauff, 1983; **Location:** country: United States; countryCode: US; stateProvince: Pennsylvania; county: Centre; verbatimCoordinates: 41.030522, -77.98226; decimalLatitude: 41.030522; decimalLongitude: -77.98226; georeferenceProtocol: label; **Event:** samplingProtocol: Lindgren multifunnel trap; eventDate: 2012-5-18; **Record Level:** language: en; institutionCode: USNM; basisOfRecord: PreservedSpecimen
- **Type status:** Other material. **Occurrence:** recordedBy: Rick Malak; individualCount: 1; lifeStage: adult; **Taxon:** scientificName: *Orussus minutus* Middlekauff, 1983; kingdom: Animalia; phylum: Arthropoda; class: Insecta; order: Hymenoptera; family: Orussidae; genus: Orussus; specificEpithet: minutus; scientificNameAuthorship: Middlekauff, 1983; **Location:** country: United States; countryCode: US; stateProvince: Pennsylvania; county: Bradford; verbatimCoordinates: 41.81719, -76.79818; decimalLatitude: 41.81719; decimalLongitude: -76.79818; georeferenceProtocol: label; **Event:** samplingProtocol: Lindgren multifunnel trap; eventDate: 2012-5-31; **Record Level:** language: en; institutionCode: USNM; basisOfRecord: PreservedSpecimen
- **Type status:** Other material. **Occurrence:** recordedBy: Sandra Gardosik; individualCount: 7; lifeStage: adult; **Taxon:** scientificName: *Orussus minutus* Middlekauff, 1983; kingdom: Animalia; phylum: Arthropoda; class: Insecta; order: Hymenoptera; family: Orussidae; genus: Orussus; specificEpithet: minutus; scientificNameAuthorship: Middlekauff, 1983; **Location:** country: United States; countryCode: US; stateProvince: Pennsylvania; county: Delaware; verbatimCoordinates: 39.85234, -75.40715; decimalLatitude: 39.85234; decimalLongitude: -75.40715; georeferenceProtocol: label; **Event:** samplingProtocol: Lindgren multifunnel trap; eventDate: 2011-4-19; **Record Level:** language: en; institutionCode: USNM; basisOfRecord: PreservedSpecimen
- **Type status:** Other material. **Occurrence:** recordedBy: Sandra Gardosik; individualCount: 7; lifeStage: adult; **Taxon:** scientificName: *Orussus minutus* Middlekauff, 1983; kingdom: Animalia; phylum: Arthropoda; class: Insecta; order: Hymenoptera; family: Orussidae; genus: Orussus; specificEpithet: minutus; scientificNameAuthorship: Middlekauff, 1983; **Location:** country: United States; countryCode: US; stateProvince: Pennsylvania; county: Delaware; verbatimCoordinates: 39.85225, -75.40751; decimalLatitude: 39.85225; decimalLongitude: -75.40751; georeferenceProtocol: label; **Event:** samplingProtocol: Lindgren multifunnel trap; eventDate: 2011-4-19; **Record Level:** language: en; institutionCode: USNM; basisOfRecord: PreservedSpecimen
- **Type status:** Other material. **Occurrence:** recordedBy: Sandra Gardosik; individualCount: 4; lifeStage: adult; **Taxon:** scientificName: *Orussus minutus* Middlekauff, 1983; kingdom: Animalia; phylum: Arthropoda; class: Insecta; order: Hymenoptera; family: Orussidae; genus: Orussus; specificEpithet: minutus; scientificNameAuthorship: Middlekauff, 1983; **Location:** country: United States; countryCode: US; stateProvince: Pennsylvania; county: Delaware; verbatimCoordinates: 39.85206, -75.40721; decimalLatitude: 39.85206; decimalLongitude: -75.40721; georeferenceProtocol: label; **Event:** samplingProtocol: Lindgren multifunnel trap; eventDate: 2011-4-19; **Record Level:** language: en; institutionCode: USNM; basisOfRecord: PreservedSpecimen
- **Type status:** Other material. **Occurrence:** recordedBy: Scott Robert; individualCount: 4; lifeStage: adult; **Taxon:** scientificName: *Orussus minutus* Middlekauff, 1983; kingdom: Animalia; phylum: Arthropoda; class: Insecta; order: Hymenoptera; family: Orussidae; genus: Orussus; specificEpithet: minutus; scientificNameAuthorship: Middlekauff, 1983; **Location:** country: United States; countryCode: US; stateProvince: Pennsylvania; county: York; verbatimCoordinates: 40.0295, -76.70635; decimalLatitude: 40.0295; decimalLongitude: -76.70635; georeferenceProtocol: label; **Event:** samplingProtocol: Lindgren multifunnel trap; eventDate: 2011-4-7; **Record Level:** language: en; institutionCode: USNM; basisOfRecord: PreservedSpecimen
- **Type status:** Other material. **Occurrence:** recordedBy: Scott Robert; individualCount: 2; lifeStage: adult; **Taxon:** scientificName: *Orussus minutus* Middlekauff, 1983; kingdom: Animalia; phylum: Arthropoda; class: Insecta; order: Hymenoptera; family: Orussidae; genus: Orussus; specificEpithet: minutus; scientificNameAuthorship: Middlekauff, 1983; **Location:** country: United States; countryCode: US; stateProvince: Pennsylvania; county: York; verbatimCoordinates: 40.03012, -76.70447; decimalLatitude: 40.03012; decimalLongitude: -76.70447; georeferenceProtocol: label; **Event:** samplingProtocol: Lindgren multifunnel trap; eventDate: 2011-5-22; **Record Level:** language: en; institutionCode: USNM; basisOfRecord: PreservedSpecimen
- **Type status:** Other material. **Occurrence:** recordedBy: L. Donovall; individualCount: 9; lifeStage: adult; **Taxon:** scientificName: *Orussus minutus* Middlekauff, 1983; kingdom: Animalia; phylum: Arthropoda; class: Insecta; order: Hymenoptera; family: Orussidae; genus: Orussus; specificEpithet: minutus; scientificNameAuthorship: Middlekauff, 1983; **Location:** country: United States; countryCode: US; stateProvince: Pennsylvania; county: Franklin; verbatimCoordinates: 39.93079, -77.63713; decimalLatitude: 39.93079; decimalLongitude: -77.63713; georeferenceProtocol: label; **Event:** samplingProtocol: Lindgren multifunnel trap; eventDate: 2011-5-2; **Record Level:** language: en; institutionCode: USNM; basisOfRecord: PreservedSpecimen
- **Type status:** Other material. **Occurrence:** recordedBy: L. Donovall; individualCount: 2; lifeStage: adult; **Taxon:** scientificName: *Orussus minutus* Middlekauff, 1983; kingdom: Animalia; phylum: Arthropoda; class: Insecta; order: Hymenoptera; family: Orussidae; genus: Orussus; specificEpithet: minutus; scientificNameAuthorship: Middlekauff, 1983; **Location:** country: United States; countryCode: US; stateProvince: Pennsylvania; county: Franklin; verbatimCoordinates: 39.93071, -77.63803; decimalLatitude: 39.93071; decimalLongitude: -77.63803; georeferenceProtocol: label; **Event:** samplingProtocol: Lindgren multifunnel trap; eventDate: 2011-5-2; **Record Level:** language: en; institutionCode: USNM; basisOfRecord: PreservedSpecimen
- **Type status:** Other material. **Occurrence:** recordedBy: L. Donovall; individualCount: 2; lifeStage: adult; **Taxon:** scientificName: *Orussus minutus* Middlekauff, 1983; kingdom: Animalia; phylum: Arthropoda; class: Insecta; order: Hymenoptera; family: Orussidae; genus: Orussus; specificEpithet: minutus; scientificNameAuthorship: Middlekauff, 1983; **Location:** country: United States; countryCode: US; stateProvince: Pennsylvania; county: Franklin; verbatimCoordinates: 39.93071, -77.63803; decimalLatitude: 39.93071; decimalLongitude: -77.63803; georeferenceProtocol: label; **Event:** samplingProtocol: Lindgren multifunnel trap; eventDate: 2011-5-1; **Record Level:** language: en; institutionCode: USNM; basisOfRecord: PreservedSpecimen
- **Type status:** Other material. **Occurrence:** recordedBy: L. Donovall; individualCount: 1; lifeStage: adult; **Taxon:** scientificName: *Orussus minutus* Middlekauff, 1983; kingdom: Animalia; phylum: Arthropoda; class: Insecta; order: Hymenoptera; family: Orussidae; genus: Orussus; specificEpithet: minutus; scientificNameAuthorship: Middlekauff, 1983; **Location:** country: United States; countryCode: US; stateProvince: Pennsylvania; county: Franklin; verbatimCoordinates: 39.930837, -77.638226; decimalLatitude: 39.930837; decimalLongitude: -77.638226; georeferenceProtocol: label; **Event:** samplingProtocol: Lindgren multifunnel trap; eventDate: 2011-5-2; **Record Level:** language: en; institutionCode: USNM; basisOfRecord: PreservedSpecimen
- **Type status:** Other material. **Occurrence:** recordedBy: L. Donovall; individualCount: 1; lifeStage: adult; **Taxon:** scientificName: *Orussus minutus* Middlekauff, 1983; kingdom: Animalia; phylum: Arthropoda; class: Insecta; order: Hymenoptera; family: Orussidae; genus: Orussus; specificEpithet: minutus; scientificNameAuthorship: Middlekauff, 1983; **Location:** country: United States; countryCode: US; stateProvince: Pennsylvania; county: Franklin; verbatimCoordinates: 39.930785, -77.637101; decimalLatitude: 39.930785; decimalLongitude: -77.637101; georeferenceProtocol: label; **Event:** samplingProtocol: Lindgren multifunnel trap; eventDate: 2011-5-2; **Record Level:** language: en; institutionCode: USNM; basisOfRecord: PreservedSpecimen
- **Type status:** Other material. **Occurrence:** recordedBy: L. Donovall; individualCount: 1; lifeStage: adult; **Taxon:** scientificName: *Orussus minutus* Middlekauff, 1983; kingdom: Animalia; phylum: Arthropoda; class: Insecta; order: Hymenoptera; family: Orussidae; genus: Orussus; specificEpithet: minutus; scientificNameAuthorship: Middlekauff, 1983; **Location:** country: United States; countryCode: US; stateProvince: Pennsylvania; county: Franklin; verbatimCoordinates: 39.930785, -77.637101; decimalLatitude: 39.930785; decimalLongitude: -77.637101; georeferenceProtocol: label; **Event:** samplingProtocol: Lindgren multifunnel trap; eventDate: 2011-8-1; **Record Level:** language: en; institutionCode: USNM; basisOfRecord: PreservedSpecimen
- **Type status:** Other material. **Occurrence:** recordedBy: L. Donovall; individualCount: 1; lifeStage: adult; **Taxon:** scientificName: *Orussus minutus* Middlekauff, 1983; kingdom: Animalia; phylum: Arthropoda; class: Insecta; order: Hymenoptera; family: Orussidae; genus: Orussus; specificEpithet: minutus; scientificNameAuthorship: Middlekauff, 1983; **Location:** country: United States; countryCode: US; stateProvince: Pennsylvania; county: Franklin; verbatimCoordinates: 39.930837, -77.638226; decimalLatitude: 39.930837; decimalLongitude: -77.638226; georeferenceProtocol: label; **Event:** samplingProtocol: Lindgren multifunnel trap; eventDate: 2011-5-2; **Record Level:** language: en; institutionCode: USNM; basisOfRecord: PreservedSpecimen
- **Type status:** Other material. **Occurrence:** recordedBy: L. Donovall; individualCount: 1; lifeStage: adult; **Taxon:** scientificName: *Orussus minutus* Middlekauff, 1983; kingdom: Animalia; phylum: Arthropoda; class: Insecta; order: Hymenoptera; family: Orussidae; genus: Orussus; specificEpithet: minutus; scientificNameAuthorship: Middlekauff, 1983; **Location:** country: United States; countryCode: US; stateProvince: Pennsylvania; county: Franklin; verbatimCoordinates: 39.930884, -77.637928; decimalLatitude: 39.930884; decimalLongitude: -77.637928; georeferenceProtocol: label; **Event:** samplingProtocol: Lindgren multifunnel trap; eventDate: 2011-4-28; **Record Level:** language: en; institutionCode: USNM; basisOfRecord: PreservedSpecimen
- **Type status:** Other material. **Occurrence:** recordedBy: L. Donovall; individualCount: 1; lifeStage: adult; **Taxon:** scientificName: *Orussus minutus* Middlekauff, 1983; kingdom: Animalia; phylum: Arthropoda; class: Insecta; order: Hymenoptera; family: Orussidae; genus: Orussus; specificEpithet: minutus; scientificNameAuthorship: Middlekauff, 1983; **Location:** country: United States; countryCode: US; stateProvince: Pennsylvania; county: Franklin; verbatimCoordinates: 39.930884, -77.637928; decimalLatitude: 39.930884; decimalLongitude: -77.637928; georeferenceProtocol: label; **Event:** samplingProtocol: Lindgren multifunnel trap; eventDate: 2011-4-21; **Record Level:** language: en; institutionCode: USNM; basisOfRecord: PreservedSpecimen
- **Type status:** Other material. **Occurrence:** recordedBy: L. Donovall; individualCount: 1; lifeStage: adult; **Taxon:** scientificName: *Orussus minutus* Middlekauff, 1983; kingdom: Animalia; phylum: Arthropoda; class: Insecta; order: Hymenoptera; family: Orussidae; genus: Orussus; specificEpithet: minutus; scientificNameAuthorship: Middlekauff, 1983; **Location:** country: United States; countryCode: US; stateProvince: Pennsylvania; county: Franklin; verbatimCoordinates: 39.930884, -77.637928; decimalLatitude: 39.930884; decimalLongitude: -77.637928; georeferenceProtocol: label; **Event:** samplingProtocol: Lindgren multifunnel trap; eventDate: 2011-5-19; **Record Level:** language: en; institutionCode: USNM; basisOfRecord: PreservedSpecimen
- **Type status:** Other material. **Occurrence:** recordedBy: L. Donovall; individualCount: 1; lifeStage: adult; **Taxon:** scientificName: *Orussus minutus* Middlekauff, 1983; kingdom: Animalia; phylum: Arthropoda; class: Insecta; order: Hymenoptera; family: Orussidae; genus: Orussus; specificEpithet: minutus; scientificNameAuthorship: Middlekauff, 1983; **Location:** country: United States; countryCode: US; stateProvince: Pennsylvania; county: Franklin; verbatimCoordinates: 39.93094, -77.637133; decimalLatitude: 39.93094; decimalLongitude: -77.637133; georeferenceProtocol: label; **Event:** samplingProtocol: Lindgren multifunnel trap; eventDate: 2011-4-1; **Record Level:** language: en; institutionCode: USNM; basisOfRecord: PreservedSpecimen
- **Type status:** Other material. **Occurrence:** recordedBy: L. Donovall; individualCount: 1; lifeStage: adult; **Taxon:** scientificName: *Orussus minutus* Middlekauff, 1983; kingdom: Animalia; phylum: Arthropoda; class: Insecta; order: Hymenoptera; family: Orussidae; genus: Orussus; specificEpithet: minutus; scientificNameAuthorship: Middlekauff, 1983; **Location:** country: United States; countryCode: US; stateProvince: Pennsylvania; county: Franklin; verbatimCoordinates: 39.93094, -77.637133; decimalLatitude: 39.93094; decimalLongitude: -77.637133; georeferenceProtocol: label; **Event:** samplingProtocol: Lindgren multifunnel trap; eventDate: 2011-4-21; **Record Level:** language: en; institutionCode: USNM; basisOfRecord: PreservedSpecimen
- **Type status:** Other material. **Occurrence:** recordedBy: L. Donovall; individualCount: 1; lifeStage: adult; **Taxon:** scientificName: *Orussus minutus* Middlekauff, 1983; kingdom: Animalia; phylum: Arthropoda; class: Insecta; order: Hymenoptera; family: Orussidae; genus: Orussus; specificEpithet: minutus; scientificNameAuthorship: Middlekauff, 1983; **Location:** country: United States; countryCode: US; stateProvince: Pennsylvania; county: Franklin; verbatimCoordinates: 39.93021, -77.638025; decimalLatitude: 39.93021; decimalLongitude: -77.638025; georeferenceProtocol: label; **Event:** samplingProtocol: Lindgren multifunnel trap; eventDate: 2011-6-1; **Record Level:** language: en; institutionCode: USNM; basisOfRecord: PreservedSpecimen
- **Type status:** Other material. **Occurrence:** recordedBy: L. Donovall; individualCount: 1; lifeStage: adult; **Taxon:** scientificName: *Orussus minutus* Middlekauff, 1983; kingdom: Animalia; phylum: Arthropoda; class: Insecta; order: Hymenoptera; family: Orussidae; genus: Orussus; specificEpithet: minutus; scientificNameAuthorship: Middlekauff, 1983; **Location:** country: United States; countryCode: US; stateProvince: Pennsylvania; county: Franklin; verbatimCoordinates: 39.93021, -77.638025; decimalLatitude: 39.93021; decimalLongitude: -77.638025; georeferenceProtocol: label; **Event:** samplingProtocol: Lindgren multifunnel trap; eventDate: 2011-5-2; **Record Level:** language: en; institutionCode: USNM; basisOfRecord: PreservedSpecimen
- **Type status:** Other material. **Occurrence:** recordedBy: L. Donovall; individualCount: 1; lifeStage: adult; **Taxon:** scientificName: *Orussus minutus* Middlekauff, 1983; kingdom: Animalia; phylum: Arthropoda; class: Insecta; order: Hymenoptera; family: Orussidae; genus: Orussus; specificEpithet: minutus; scientificNameAuthorship: Middlekauff, 1983; **Location:** country: United States; countryCode: US; stateProvince: Pennsylvania; county: Franklin; verbatimCoordinates: 39.93097, -77.637695; decimalLatitude: 39.93097; decimalLongitude: -77.637695; georeferenceProtocol: label; **Event:** samplingProtocol: Lindgren multifunnel trap; eventDate: 2011-4-21; **Record Level:** language: en; institutionCode: USNM; basisOfRecord: PreservedSpecimen
- **Type status:** Other material. **Occurrence:** recordedBy: Anton Jansson; individualCount: 2; lifeStage: adult; **Taxon:** scientificName: *Orussus minutus* Middlekauff, 1983; kingdom: Animalia; phylum: Arthropoda; class: Insecta; order: Hymenoptera; family: Orussidae; genus: Orussus; specificEpithet: minutus; scientificNameAuthorship: Middlekauff, 1983; **Location:** country: United States; countryCode: US; stateProvince: Connecticut; county: New London; municipality: Groton; georeferenceProtocol: label; **Event:** eventDate: 17695.00; **Record Level:** institutionCode: MZLU; basisOfRecord: PreservedSpecimen

#### Distribution

New York south to Georgia west to Manitoba, Iowa, and Arkansas.

#### Analysis

The Arkansas specimens were identified morphologically as *Orussus minutus* Middlekauff, 1983 (Figs 1, 2). The 16S rRNA sequence (GenBank #KM379143) was a 99.5% match with an existing *O. minutus* sequence (EF032174), differing by two base pairs.

## Discussion

The Arkansas specimens and those shared as photographs on Bugguide and Flickr significantly expand the known range of *O. minutus* westward (Fig. 3). Morphological determination of the Arkansas specimens was confirmed by genetic data and the species is easily identified due to its small size and distinct markings, so it is highly unlikely the photographed specimens are not *O. minutus*.

Many of the USNM specimens were collected by David R. Smith during 35 years of Malaise trapping specifically for sawflies. However, most recently collected specimens, especially those from Pennsylvania, were found as non-target species during various exotic species monitoring programs that utalized Lindgren multifunnel traps (David Smith, pers. comm., 28 Aug. 2015). The abundance of these specimens emphasize the utility of examining, or at least collecting and sending to the appropriate specialist, non-target species in mass trapping surveys, such as was suggested by Skvarla and Holland (2011). Precise figures for the number of traps and amount of effort that was involved in the Pennsylvania surveys is unavailable, so we are unable to compare the efficiency of Malaise trapping compared to Lindgren funnel trapping; however, the number of *O. minutus* that were collected in Lindgren funnel traps suggests that it may be a useful tool for collecting *Orussus*.

Given the current records, *O. minutus* is likely present throughout most of Eastern North America. The concentration of specimens from northern Virginia and Pennsylvania reflect collecting effort and specimen recoginition rather than true abundance and further collecting in the southeastern United States and Canada should produce additional specimens from those areas.

Finally, records found through Bugguide and Flickr join a growing list of discoveries made via citizen science and social media websites (e.g., Otto and Hill 2011, Winterton et al. 2012, Gonella et al. 2015) and help underscore the importance of such resources in descriptive biology and natural history.

## Supplementary Material

XML Treatment for Ourssus minutus

## Figures and Tables

**Figure 1a.**
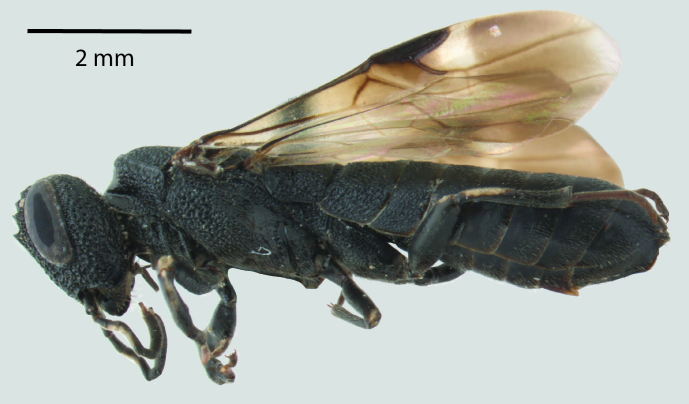
Lateral habitus

**Figure 1b.**
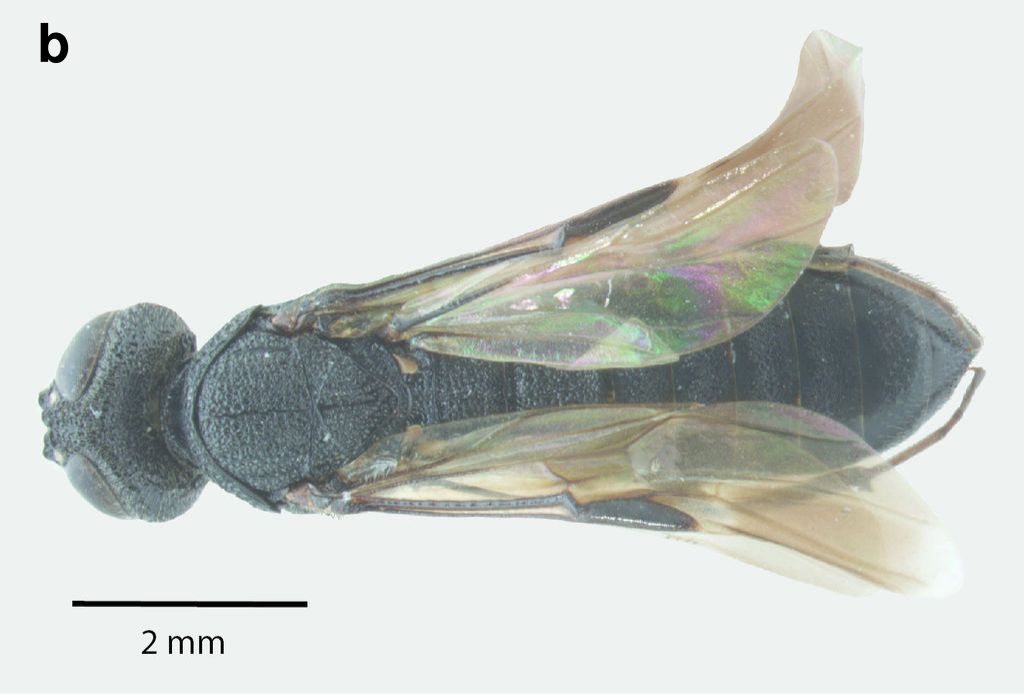
Dorsal habitus

**Figure 1c.**
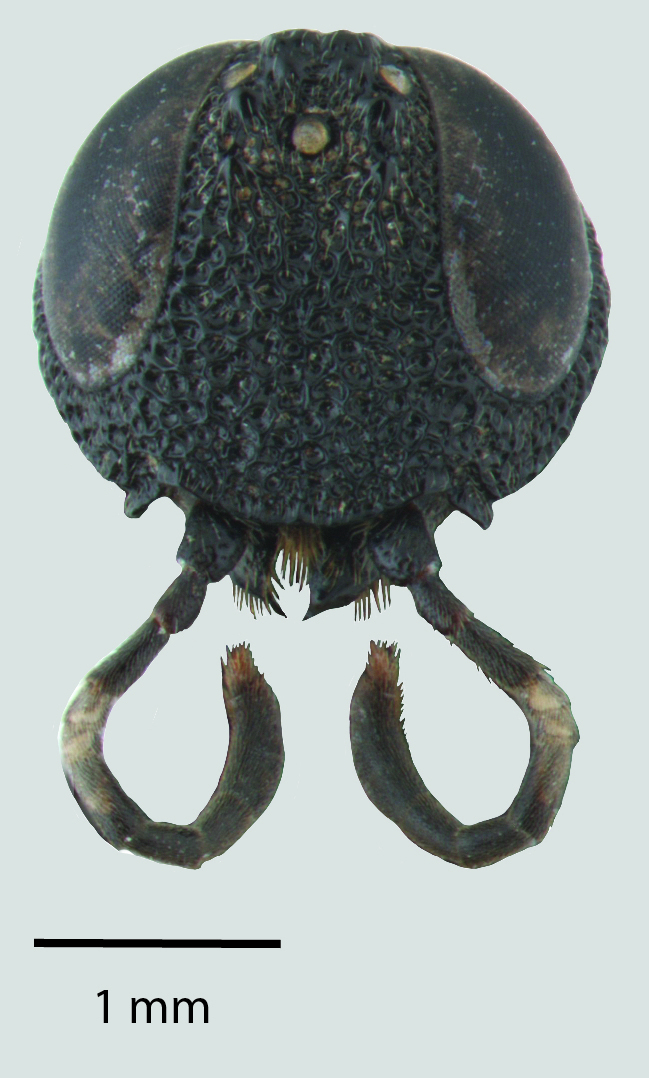
Head

**Figure 1d.**
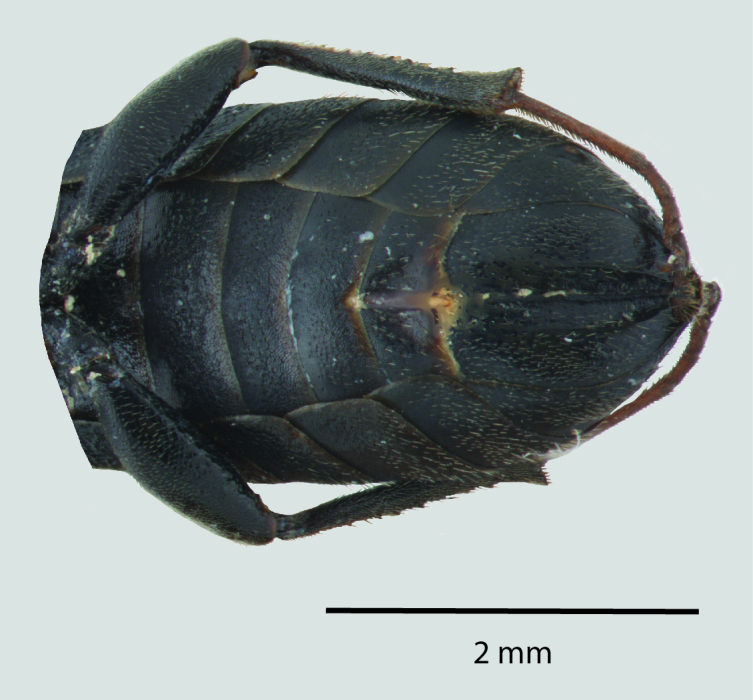
Ventral abdomen

**Figure 2a.**
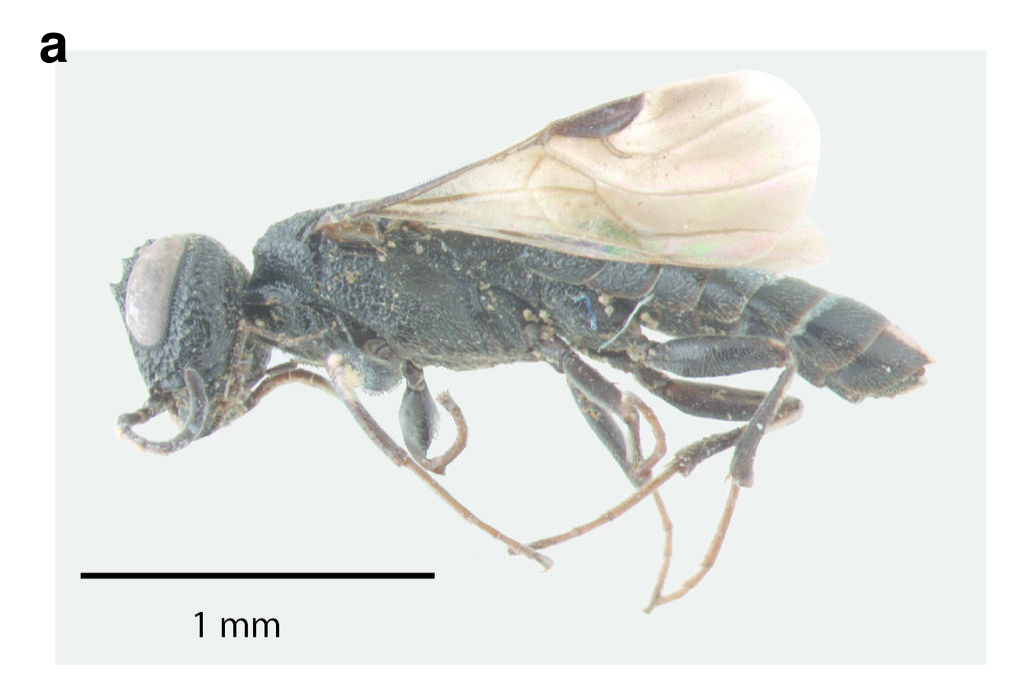
Lateral habitus

**Figure 2b.**
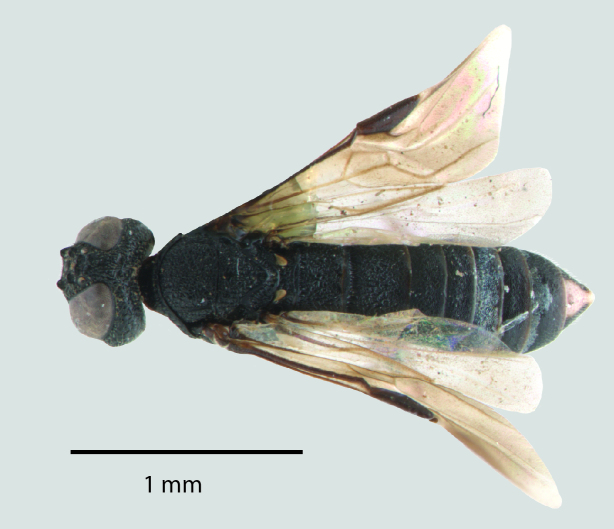
Dorsal habitus

**Figure 2c.**
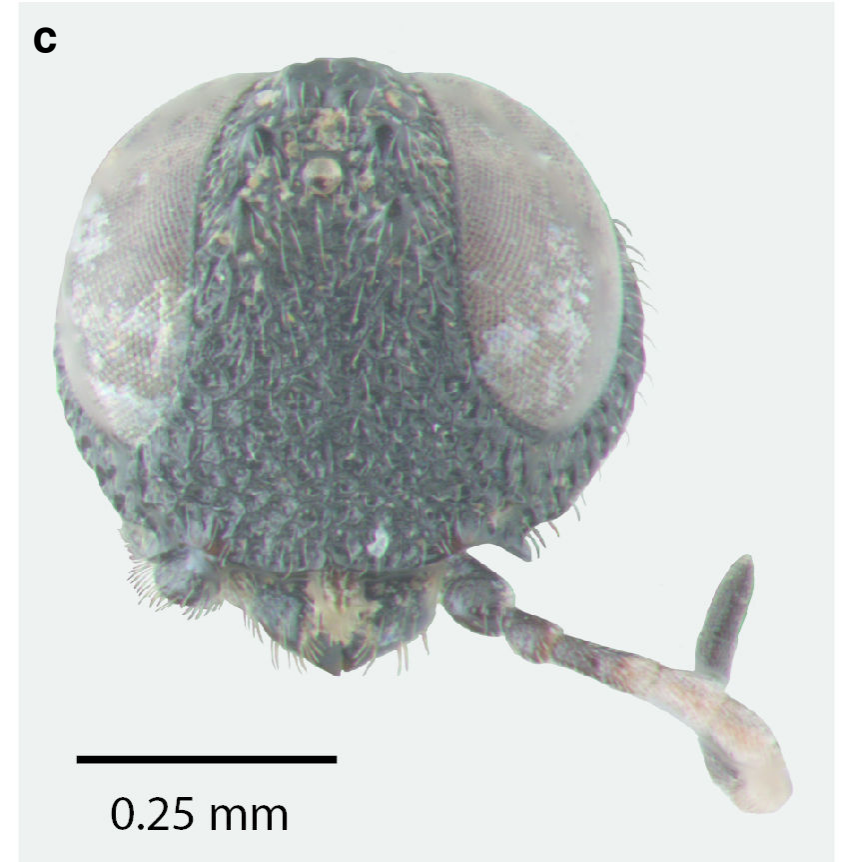
Head

**Figure 2d.**
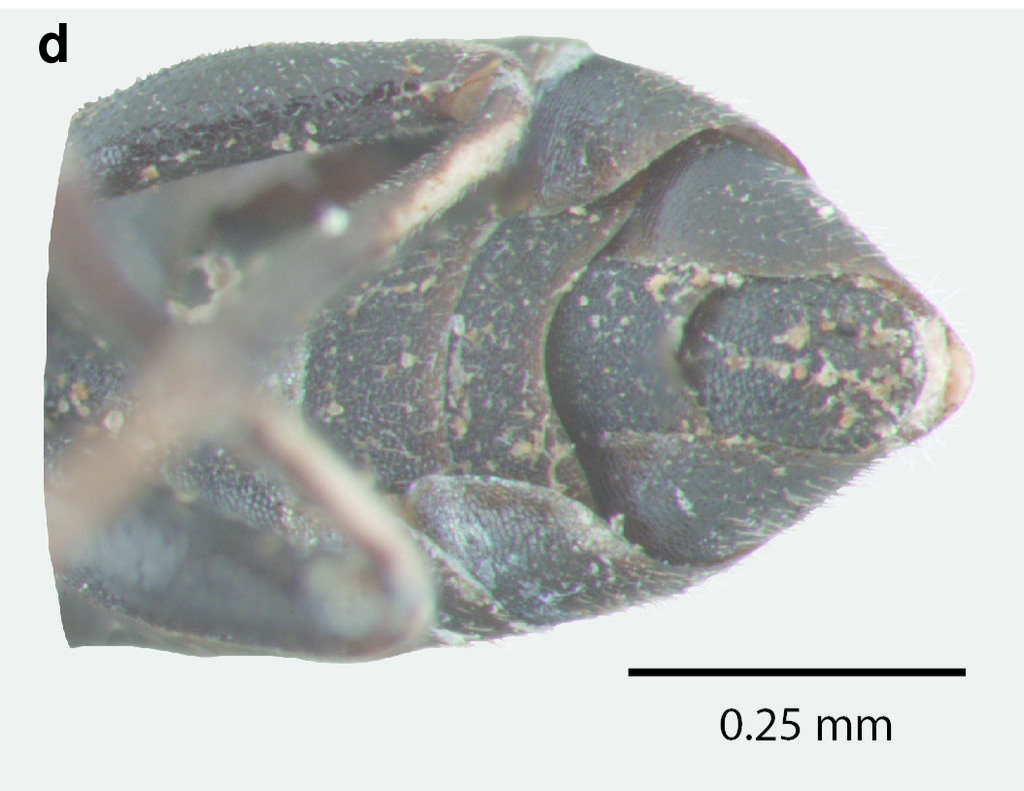
Ventral abdomen

**Table 1.** Known and suspected hosts of Orussidae

**Host family**	**Reference**
Buprestidae	Wachtl 1882, Harrington 1887a, Burke 1918, [Bibr B1665189][Bibr B1665283], Harrington 1887a, Burke 1918, Powell and Turner 1975, Ahnlund and Ronquist 2001, Vilhelmsen and Smith 2002
Cerambycidae	Rohwer 1912, Powell and Turner 1975, Hellrigl 1984, Ahnlund and Ronquist 2001
Siricidae	Gourley 1951, Rawlings 1957, Powell and Turner 1975, Vilhelmsen and Smith 2002
Xiphydriidae	Rudow 1909
